# The role and potential of using quantitative MRI biomarkers for imaging guidance in brain cancer radiotherapy treatment planning: A systematic review

**DOI:** 10.1016/j.phro.2023.100476

**Published:** 2023-07-25

**Authors:** Abeer M. Aldawsari, Bashar Al-Qaisieh, David A. Broadbent, David Bird, Louise Murray, Richard Speight

**Affiliations:** a Leeds Institute of Cardiovascular & Metabolic Medicine (LICAMM), University of Leeds, Woodhouse, Leeds LS2 9JT, United Kingdom; b Radiological Sciences Department, College of Applied Medical Sciences, King Saud University, Riyadh 12371, Saudi Arabia; c Department of Medical Physics and Engineering, Leeds Teaching Hospitals NHS Trust, Leeds LS9 7TF, United Kingdom; d Department of Clinical Oncology, Leeds Teaching Hospitals NHS Trust, St James’s University Hospital, Leeds LS9 7LP, United Kingdom; e Leeds Institute of Medical Research, University of Leeds, Leeds LS2 9JT, United Kingdom

**Keywords:** Quantitative MRI biomarker, Radiotherapy planning, Brain tumor, Target volume delineation, Systematic review

## Abstract

**Background and purpose:**

Improving the accuracy of brain tumour radiotherapy (RT) treatment planning is important to optimise patient outcomes. This systematic review investigates primary studies providing clinical evidence for the integration of quantitative magnetic resonance imaging (qMRI) biomarkers and MRI radiomics to optimise brain tumour RT planning.

**Materials and methods:**

PubMed, Scopus, Embase and Web of Science databases were searched for all years until June 21, 2022. The search identified original articles demonstrating clinical evidence for the use of qMRI biomarkers and MRI radiomics for the optimization of brain cancer RT planning. Relevant information was extracted and tabulated, including qMRI metrics and techniques, impact on RT plan optimization and changes in target and normal tissue contouring and dose distribution.

**Results:**

Nineteen articles met the inclusion criteria. Studies were grouped according to the qMRI biomarkers into: 1) diffusion-weighted imaging (DWI) and perfusion-weighted imaging (PWI; five studies); 2) diffusion tensor imaging (DTI; seven studies); and 3) MR spectroscopic imaging (MRSI; seven studies). No relevant MRI-based radiomics studies were identified. Integration of DTI maps offers the potential for improved organs at risk (OAR) sparing. MRSI metabolic maps are a promising technique for improving delineation accuracy in terms of heterogeneity and infiltration, with OAR sparing. No firm conclusions could be drawn regarding the integration of DWI metrics and PWI maps.

**Conclusions:**

Integration of qMRI metrics into RT planning offers the potential to improve delineation and OAR sparing. Clinical trials and consensus guidelines are required to demonstrate the clinical benefits of such approaches.

## 1 Introduction

Brain and central nervous system (CNS) tumours are the tenth leading cause of mortality among cancer patients in the UK [1]. In 2020, globally, approximately 308,000 new brain and CNS cancer cases were reported, accounting for 1.6% of all cancer cases, and around 252,000 deaths occurred (2.5% of total cancer deaths) [2]. The standard treatment approaches for primary brain tumours may vary depending on the type of tumour, its stage and the overall health of the patient. Typically, the initial approach for high-grade glioma (HGG), the most common adult primary malignant brain tumour, is surgical resection, followed by high-dose radiotherapy (RT) (+/-concomitant chemotherapy, followed by adjuvant chemotherapy). Where treatment of brain metastases is highly individualised, and can be treated with surgery, stereotactic radiosurgery (SRS) and whole-brain RT. Radiotherapy aims to deliver the optimal radiation dose to the target while sparing organs at risk (OAR). Optimized imaging is a critical part of the treatment pathway in achieving this.

Anatomical magnetic resonance imaging (MRI) is an essential part of the radiotherapy treatment pathway when treating patients with brain tumours, given its improved soft tissue contrast resolution compared to Computed Tomography (CT) [3]. MRI therefore enables the precise delineation of target volumes (TVs) and OAR, thereby reducing target delineation uncertainties. A range of MRI techniques could be incorporated into RT to prevent tumour recurrence [4], improve tumour detection and grading [5], enhance TVs delineation [6] and support response and prognosis monitoring [7]. However, anatomical MRI can only provide information on macrostructural morphology, whereas functional MRI techniques can identify pathophysiological tissue changes.

Advanced MRI techniques can provide functional and quantitative imaging biomarkers for microscopic assessment of the expansion of tumour infiltration to improve TVs definition accuracy [8]. Several functional MRI techniques, including diffusion-weighted imaging (DWI), diffusion-tensor imaging (DTI), perfusion-weighted imaging (PWI), MR spectroscopic imaging (MRSI) and blood oxygenation level-dependent functional MRI (BOLD-fMRI) are widely used in brain tumour assessment and may also be integrated into RT planning. Integrating more than two functional and anatomical MRI techniques to characterise tumour heterogeneity is termed multiparametric MRI (mpMRI) [9]. These advanced MRI techniques can generate quantitative MRI biomarkers to potentially enhance brain TVs edge detection and delineation accuracy.

Apparent diffusion coefficient (ADC) is the most common DWI metric and describes the magnitude of diffusion of free water molecules to evaluate tumour cellularity [10]. DTI requires more diffusion directions than DWI; a minimum of six non-colinear diffusion-encoding directions are needed to produce directional mapping of water diffusion [11]. DTI can measure the quantitative DTI maps including, fractional anisotropy (FA) maps, anisotropy (q) and isotropic (p) diffusion maps and three-dimensional fiber tracking (3D-FT) maps [12]. DTI data can also be analysed to generate the trajectory of white matter (WM) fibre tracts to form tractography maps [13].

The most common PWI techniques in brain oncology are dynamic contrast enhancement (DCE-MRI) and dynamic susceptibility contrast (DSC-MRI) [14]. These techniques act as tumour angiogenesis biomarkers to determine hemodynamic properties and vascular permeability by analysing the transit of injected contrast agents [15]. The main quantitative PWI metrics include relative cerebral blood volume (rCBV) and relative cerebral blood flow (rCBF) [16].

Three-dimensional (3D) proton MRSI estimates tumour activity by measuring the cellular metabolite concentrations in brain tissues [17]. Choline (Cho) to N-acetyl-aspartate (NAA) ratio is the most common parameter to evaluate malignant brain tumours [18]. BOLD-fMRI evaluates deoxyhemoglobin concentration based on neuronal activity changes during normal physiological activities [19] to develop brain activation maps that aid in accurate tumour risk assessment.

Radiomics in the medical imaging field focuses on extracting quantitative tumour features/metrics within images such as MRI images. These features include, for example, shape characteristics, texture patterns and intensity values. These are then analysed using mathematical algorithms to quantify imaging biomarkers that provide mineable high-dimensional data on tumour cells [20]. Radiomics has the potential to offer tumours pathophysiological information, thereby improving diagnostic decisions and guiding oncological RT planning [5]. Therefore, integrating quantitative MRI biomarkers and MRI-based radiomics into the brain tumour RT pathway, could further improve RT planning.

A recent systematic review reported the clinical benefits of integrating DTI into intracranial RT planning to optimise WM dose [21]. However, no other systematic reviews have established clinical evidence for the role of various quantitative MRI biomarkers and MRI radiomics in brain cancer RT planning. This review aims to critically and systematically evaluate primary studies reporting clinical evidence about the use of quantitative MRI biomarkers and MRI radiomics to enhance brain tumour RT planning.

## 2 Material and methods

### 2.1 Search strategy

A systematic review was carried out using the Preferred Reporting Items for Systematic Reviews and Meta-Analyses (PRISMA) guidelines [22]. The Embase, PubMed, Scopus and Web of Science databases were searched with a time span of all years to June 21, 2022, for full-text and primary research articles in the English language published in peer-reviewed journals for all study designs. The search terms were: “MRI OR mpMRI” AND “radiotherapy” AND “radiomics OR texture OR quantitative OR biomarkers” AND “brain tumours” or synonyms of these terms in the title or abstract. Search criteria examples are presented in Table 1. These broad search criteria were employed to minimise the risk of missing relevant studies. All review articles, books and grey literature were excluded as these were not considered beneficial to this review.

**Table 1:** Example of two databases (PubMed and Web of Science), search terms and criteria.

Databases	#	Search terms
PubMed	1	(“Magnetic Resonance Imaging”[MeSH Terms] OR “Multiparametric Magnetic Resonance Imaging”[MeSH Terms] OR “MRI”[Title/Abstract] OR “multiparametric mr*”[Title/Abstract] OR “magnetic resonance*”[Title/Abstract])
	2	(“radiomic*”[Title/Abstract] OR “texture”[Title/Abstract] OR “textural”[Title/Abstract] OR “quantitative*”[Title/Abstract] OR “biomarker*”[Title/Abstract])
	3	(“radiotherapy”[MeSH Terms] OR “radiotherap*”[Title/Abstract] OR “radiation therap*”[Title/Abstract] OR “radiation oncology”[Title/Abstract] OR “radiation treatment*”[Title/Abstract])
	4	(“Brain Neoplasms”[MeSH Terms] OR “brain tumor*”[Title/Abstract] OR “brain cancer*”[Title/Abstract] OR “brain neoplasm*”[Title/Abstract] OR “Glioblastoma”[Title/Abstract] OR “glioma*”[Title/Abstract] OR “GBM”[Title/Abstract] OR “meningioma*”[Title/Abstract] OR “brain metastas*”[Title/Abstract] OR “brain malignan*”[Title/Abstract] OR “intracranial tumor*”[Title/Abstract])
	5	#1 AND #2 AND #3 AND #4 AND (English [Filter])
Web of Science	1	(TS= (MRI OR “Magnetic Resonance Imaging” OR “multiparametric mr*” OR “Magnetic resonance*”)
	2	TS= (radiomic? OR texture OR textural OR quantitative* or biomarker?)
	3	TS= (Radiotherapy OR radiotherap* OR “radiation therap*” OR “radiation oncology” OR “radiation treatment*”)
	4	TS= (“Brain tumo?r*” OR “Brain neoplasm*” OR Glioblastoma OR Glioma* OR GBM OR meningioma? OR “brain malignan*” OR “Brain metastas*” OR “brain cancer*” OR “intracranial tumo?r*”)
	5	#1 AND #2 AND #3 AND #4 AND (DT==(“ARTICLE”) AND LA==(“ENGLISH”))

**Abbreviations:** DT = document type; LA = language; MeSH = medical subject headings; MRI = Magnetic Resonance Imaging TS = Searches for topic terms in the following fields within a record: title, abstract, author keywords and keywords plus.

### 2.2 Study selection

**Fig. 1:**
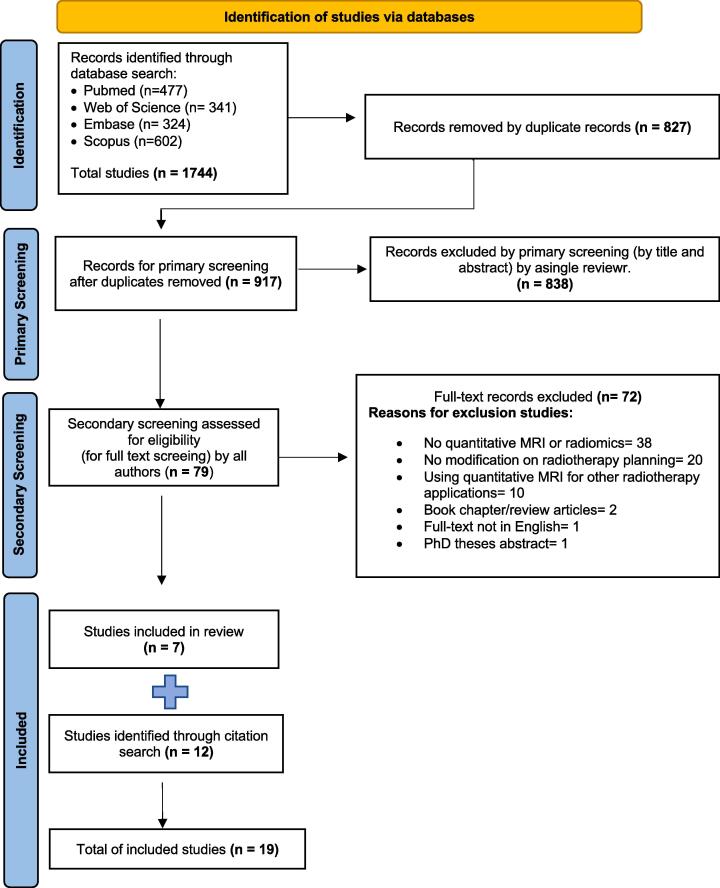
The Preferred Reporting Items for Systematic Reviews and Meta-Analyses (PRISMA) flow diagram. This Flowchart addressed the systematic review process based on PRISMA guidelines, for 19 inclusion studies in this review.

After combining search results for each database, duplicate articles were removed using Endnote^x9^ (Endnote X9.3.3, Clarivate^TM^, Philadelphia, PA). The remaining articles were screened for eligibility, primarily based on the title and abstract, then full-text screening, according to inclusion and exclusion criteria using Rayyan [23]; detailed search results in each stage are shown in Fig. 1.

#### 2.2.1 Inclusion criteria

Relevant articles were considered those that reported clinical evidence regarding the use of quantitative data from mpMRI or MRI radiomics in the RT planning pathway for brain tumour patients of all ages.

#### 2.2.2 Exclusion criteria

The following were excluded:

• Studies focusing on other applications of MRI in radiotherapy, such as prediction, response assessment, tumour progression, radiation injury, tumour segmentation or classification;
• Studies comparing MRI to synthetic CT or positron emission tomography, not reporting quantitative MRI metrics, employing qualitative/conventional MRI in RT planning, or assessing the use of radiomics algorithms for surgery planning; and
• Studies about quantitative MRI biomarkers or MRI radiomics that were not used to optimise RT planning.

The secondary screening reviewed the full text based on the inclusion and exclusion criteria. A forward and backward citation search of identified research articles.

### 2.3 Data extraction and synthesis

The relevant information from each eligible included study was extracted into Excel (Office 365, Microsoft Corporation, Washington, USA). This spreadsheet included the key findings from published planning studies and included authors, publication year, study type (prospective or retrospective), sample size, RT types, brain cancer types, MRI scanner features, MRI techniques and acquisition, and MRI-derived quantitative metric/maps and thresholds. Changes in target or OAR contouring, dosimetric and volumetric evaluation, RT planning optimization, technical limitations and clinical findings were also recorded. Any unclear or missing information was reported as “unclear” or “not reported” in the data table. The data synthesis involved thematic analysis based on quantitative MRI techniques used in studies.

## 3 Results

### 3.1 Search strategy

A flowchart of database search results is shown in Fig. 1. The combined database search yielded 1744 records, with 917 remaining after removing duplications. After primary screening using Rayyan by a single reviewer, 79 studies underwent full-text review by all authors. Among them, 19 articles exhibited discrepancies in authors’ assessments. Of these, 72 articles did not fulfil the eligibility criteria. Seven studies fulfilled the inclusion criteria, and an additional 12 articles were identified from the citation search, with inclusion agreed by all authors, giving a total of 19 articles. The additional articles were likely missed in the original search because the title and abstract did not contain the terms “quantitative OR biomarkers OR radiomics OR texture”.

### 3.2 Study characteristics

Table 2 summarises the study characteristics of 19 eligible articles. Based on the quantitative MRI techniques used to guide RT planning, included studies were classified as follows:

1. DWI and PWI [24], [25], [26], [27], [28];
2. DTI [29], [30], [31], [32], [33], [34], [35]; and
3. MRSI [36], [37], [38], [39], [40], [41], [42].

**Table 2:** Characteristics of included studies and summary of the key results.

#	Reference	Number of patients	Type of RT/RS planning	Cancer type	MRI scanner (Magnetic strength, Tesla) (Model, vendor; coil)	Functional/anatomical MRI techniques	Changing TV/OAR contouring	Treatment plans optimisation	Radiation dose changes	Target volume changes
**DWI**										
**1**	[24]	1	IMRT	HGG	**1.5T** (Signa, GE)	DWI PWI MRSI T1-CE	CTV Improve dose sparing to OAR	Yes	Reduce dose to OAR and improve dose to CTV_HR_	NR
**2**	[25]	17	SRS	Brain metastases	**1.5T** (single channel head coil)	DWI 3D T1-CE	GTV	Volumetric analysis of different GTVs	NR	Increase
**DWI & PWI**										
**3**	[26]	12	NR	GBM	**3.0T** (Skyra, Siemens; 20-channel head coil)	High b-value DWI DCE T1-quantification T1-CE T2-FLAIR	GTV Maintaining dose limits to OAR	Yes	Boost volume for dose-intensified RT	Decrease
**4**	[27]	26	NR	GBM Gliosarcoma	**3.0T** (Skyra, Siemens; 20-channel head coil)	High b-value DWI DCE 3D T1-CE T2-FLAIR	NR	Yes	dose-intensified RT	NR
**5**	[28]	11	IMRT	GBM	NR (multisite)	DWI DSC T1-CE T2 FLAIR	NR	Repeatability of mpMRI metrics derived DP maps and TP maps	NA	NA
**DTI**										
**6**	[29]	7	3D-CRT	HGG	**3.0T** (Bruker BioSpec, Ettlingen)	DTI	CTV & PTV maintain NTCP dose	Yes	Improve dose to DTI-based plan	Smaller CTV_DTI_ & PTV_DTI_
**7**	[30]	13	IMRT	GBM	**1.5T** (Avanto, Siemens; 12-channel head coil	DTI	GTV + CTV	Yes	Limit dose to uninvolved brain tissues	Smaller CTV_DTI_
**8**	[31]	NR	IMRT	GBM/HGG	**1.5T** (head coil)	DTI	Improve dose constrains to CSTs and other OAR	Yes	DTI-based reduce dose to CSTs and other OAR	NC
**9**	[32]	20	3D-CRT IMRT	HGG	**3.0 T** (Signa HDx, GE)	DTI BOLD-fMRI	Dose reduction to PMCs & CSTs	Developed 3 plans for comparisons	Reduce max dose to the ipsilateral and contralateral PMCs and CSTs regions	NC
**10**	[33]	11	NR	GBM	**1.5 T** (Ingenia, Philips; brain coil)	DTI	CTV surface volume	Yes	NC	Increase
**11**	[34]	19	HT	HGG	**3.0T**= 4 pt **1.5T=** 15 pt; (Achieva, Philips) **(multscanners)**	DTI	Dose sparing to WM fibres tracts (SLF, IFOF, UNC and CSTs)	Yes	Tract-optimized plan improve dose sparing to WM fibres tracts	NC
**12**	[35]	35	IMRT	HGG (nTMS motor mapping)	NR	DWI DTI-fibres tracking	Dose constrains to OAR and CST	Yes	DTI reduce dose to brain and OAR after CSTs sparing	NC
**13**	[36]	30	**27 pt;** for 3D-CRT **3 pt;** for IMRT	HGG	**1.5 T** (Signa, GE; quadrature head coil)	3D- CSI (MRSI)	CTV	Yes	NR	Estimated increase volume of CTV
**14**	[37]	23	3D-CRT	GBM/HGG	**1.5 T** (Signa, GE; quadrature head coil)	3D- MRSI	GTV	Yes	NR	GTV volume extension outside 60 Gy IDL
**15**	[38]	16	3D-CRT, IMRT and SIB_IMRT	GBM	**1.5 T** (Avanto, Siemens)	3D-CSI (MRSI)	Reducing dose to OAR	Yes	Improve target dose and decrease dose to OAR.	NR
**16**	[39]	19	NR	GBM	**3.0 T** (Signa, GE)	Whole-brain 3D-MRSI	MTVs	Yes	NR	MTV_Cho_ is larger than edema, CTV_46_ and CTV_60_
**17**	[40]	11	NR	GBM	**3.0T** (Trio, Siemens; 32-channel head coil	Whole-brain 3D MRSI	CTV Maintain OAR dose limits	Yes	NR	MRSI_CTV higher coverage
**18**	[41]	17	NR	Mutant IDH1 gliomas	**3.0T** (Trio, Siemens; 32-channel head coil	3D-MRSI (2HG imaging)	NC	Yes	NR	CTV of 2HG/hCr more volume than FLAIR
**19**	[42]	18	NR	GBM	**3.0T** (Prisma, Trio and Skyra; Siemens; 32/20-channel head coil) **(multisite)**	Whole-brain 3D MRSI	GTV Dose constraints to OAR	Yes	Improve target dose of MRSI plan	GTV of Cho/NAA abnormality has larger volume

**Abbreviation:** 2HG = 2-hydroxyglutarate; 2HG/hCr = ratio of 2-hydroxyglutarate over healthy creatine; 3D-CRT = three-dimensional conformal radiation therapy; 3D-CSI = three-dimensional chemical shift imaging; BOLD-fMRI = Blood-oxygen-level-dependent imaging – functional magnetic resonance imaging; Cho/NAA = Choline/N-Acetylaspartate (NAA) ratio; CSTs = corticospinal tracts; CTV = clinical target volume; CTV_46_ and CTV60 = Clinical target volume at dose 46 Gy and 60 Gy; CTV_HR_ = high-risk clinical target volume; DCE = dynamic contrast enhanced; DP = Dose-painting prescription maps; DSC = dynamic susceptibility contrast; DTI = Diffusion-tensor imaging; DWI = Diffusion-weighted imaging; GBM = Glioblastoma Multiforme; GE = General Electric; GM = gray matter; GTV = gross target volume; HGG = high grade glioma; HT = helical tomotherapy; IDH1 = isocitrate dehydrogenase 1; IDL = isodose line; IFOF = inferior fronto-occipital fascicle; IMRT = intensity modulated radiation therapy, MRI = Magnetic resonance imaging; MRSI = Magnetic resonance spectroscopic imaging; MTVs = metabolic tumor volumes; MTV_Cho_ = metabolic tumor volumes of high Choline; mpMRI = multiparametric MRI; NA = not applicable; NC = no changes; NR = not reported; NTCP = Normal Tissue Complication Probability; nTMS motor mapping = navigated transcranial magnetic stimulation motor mapping; pt = patients; OAR = organs at risk; PMCs = primary motor cortexes; PTV = planning target volume; PTV_HR_ = high-risk planning target volume; PWI = Perfusion-weighted imaging; RS = radiosurgery; RT = radiotherapy; SIB = simultaneous integrated boost; SLF = superior longitudinal fascicle; SRS = Stereotactic radiosurgery; T1-CE = T1-weighted contrast-enhanced; T2-FLAIR = T2-weighted Fluid attenuated inversion recovery; TP = tumour probability maps; TV = target volume; UNC = uncinate fascicle; WM = white matter.

DWI and PWI techniques were combined into a single category because three of the five studies used a combination of DWI metrics and PWI maps.

The planning studies were performed using either 1.5 T MRI (eight studies) or 3.0 T MRI (nine studies). Of these, only [42] and [28] were conducted on multi-site studies. All but one study [34] used different magnetic field strength scanners in the same study. Included studies reported different outcomes regarding the impacts of integrating functional MRI techniques on TVs or OAR in terms of volume/contouring, radiation dose, or RT plan optimisation. These studies concerned glioblastoma (GBM) [26], [27], [28], [30], [31], [33], [38], [39], [40], [41], [42], HGG [24], [29], [32], [34], [35], [36] and brain metastases [25]. Findings for each of the three MRI categories are listed in Table 3, Table 4, Table 5.

**Table 3:** Summary of Diffusion-weighted imaging and perfusion weighted imaging techniques in brain cancer radiotherapy planning.

#	Reference	Study type	Functional MRI (sequence, parameters & acquisition time)	MRI-derived quantitative metrics	qMRI post-processing availability	Threshold (range)	Modification to treatment planning	Findings of changing radiation dose/target volumes
1	[24]	NR	**DWI** (b = 0, 1,000 s/mm^2^, 3 diffusion gradients)	ADC maps	MATLAB	**Average ADC of CTV_HR_** = 0.73 × 10^-3^ s/mm^2^	**IMRT _conventional_ plan** tCTV = T1-CE + CT images (59.4 Gy) **IMRT_ADC_ plan:** aCTV_HR_ = ADC-based CTV (59.4 Gy) tCTV = tGTV + 2 cm (50.4 Gy) sCTV = aCTV_HR_ in tCTV	1. IMRT_ADC_ plan improved the dose conformity of the aCTV_HR_ up to 15 times compared to the IMRT_conv_ plan. DVHs showed greater dose sparing of OAR in the IMRT_ADC_ plan.
2	[25]	Retrospective clinical study	**DWI** (EPI, **b** = 0, 1,000 s/mm^2^, 90 s)	ADC maps	GE FuncTool software package	NR	**Volumetric analysis comparison**GTV (T1_CE) vsGTV (ADC) vs GTV (DTV) = T1-CE + ADC	1) DTV has greater volume of tumour than the GTV (T1_CE) of standard plan (median 13.65 vs. 9.52 cm^3^), respectively for SRS planning.
3	[26]	Prospective (Clinical trial - phase II)	**High b-value DWI** (2D-EPI, RESOLVE**, b** = 0 & 3000 s/mm^2^, parallel imaging of 4, 4.23 min) **DCE** (3D-GRE, dynamic phases = 60, 3 min) **T1 quantification** (3D-GRE with 4 flip angles [3°, 7°, 12°, 16°], 1.45 min)	**DCE:**hCBV **high b-value DWI:**HCV	Tofts models quantify DCE MRI parameters: V_p,_ K^trans^ and V_e_ to produce CBV	**hCBV =** 1 SD above contralateral frontal lobe GM **HCV =** 2 SD contralateral above normal tissue	**Conventional RT plan:** GTV_FLAIR = T2/FLAIR abnormalityGTV_Low = T1-CE + RC + residual CTV_Low = GTV_Low + 1.7 cm PTV_Low = 60 Gy **Advanced MRI plan** GTV_high = CBV maps + 0–3 cm CTV_high = HCV/hCBV boost volumes PTV_high = 75 Gy	1. HCV and hCBV target volumes were 1.8 times smaller than the T1-CE and 10 times smaller than FLAIR abnormality. First report of the prospective implementation of mpMRI that is integrated into RT planning.
4	[27]	Interventional study	**High b-value DWI** (2D-EPI, RESOLVE, b = 0 & 3000 s/mm^2^) DCE (3D-GRE)	**DCE:** TV _CBV_ **high b-value DWI:** TV_HCV_		**TV_CBV_** > 1 SD above contralateral frontal lobe GM **TV_HCV_** = 2 SD above the mean intensity of contralateral normal brain	**Standard target volumes** T1-CE & T2/FLAIR GTV_Low = surgical cavity and residual CE CTV_Low = 1.7 cm margin + GTV PTV_Low = 3 mm + CTV_Low, (60 Gy) **Advanced MRI plan** GTV_High = TV_HCV_ & TV_CBV_ PTV_High = 5 mm margin 75 Gy at 2.5 Gy/fraction (30 fractions)	1. Dose escalation had a significantly greater mid-radiation decrease in combined TV_HCV_/TV_CBV._ Mid-radiation growth in combined TV_HCV_/TV_CBV_ is associated with worse survival. Targeting TV_HCV_ and TV_CBV_ using MR techniques shows promise for improving outcomes.
5	[28]	Retrospective(Repeatability test–retest)	**DWI** (2D-EPI, **b** = 0 &700 s/mm^2^) **DSC** (2D-EPI, 2.41 min)	ADC & rCBV maps ADC & rCBF maps	TP and DP calculated from ADC and DSC metrics.	NA	**Standard plan:** GTV = T1-CE CTV = GTV + 2 cm **Dose-painting plans:**Volume of interest for the repeatability analysis of mpMRI-derived TP and DP maps extend from GTV to CTV	1. DP maps were repeatable for providing reliable dose-painting plans.ADC maps showed higher repeatability than rCBV/rCBF maps.TP maps were the most stable (median ICC: 0.89) . Technical validation framework for practical application of any mpMRI model.

Abbreviation: 2D-EPI = two-dimensional echo planar spin echo pulse sequence; 3D-GRE = three-dimensional gradient echo pulse sequence; ADC = apparent diffusion coefficient; CBV = cerebral blood volume; CTV = clinical target volume; CTV_HR_ = high-risk clinical target volume; DCE = dynamic contrast enhanced; DP = dose-painting prescription; DSC = dynamic susceptibility contrast; DTV = diffusion treatment volume; DVHs = dose volume histograms; EPI = echo planner imaging; FLAIR = fluid-attenuated inversion recovery; GE = general electric; GM = gray matter; GTV = gross target volume; hCBV = high cerebral blood volume; HCV = hypercellular volume; ICC = interclass correlation; IMRT = intensity modulated radiation therapy; K_trans_ = transfer constant of contrast; MRI = magnetic resonance imaging; mpMRI = multiparametric MRI; NA = not applicable; NR = not reported; OAR = organs at risk; PTV = planning target volume; qMRI = quantitative MRI; RC = resection cavity; RT = radiotherapy; rCBF = relative cerebral blood flow; rCBV = relative CBV; RESOLVE = readout segmentation of long variable echo-trains; SD = standard deviation; SRS = stereotactic radiosurgery; T1-CE = T1-weighted contrast-enhanced; TP = tumour probability; TV_CBV_ = hyperperfused tumor volume; TV_HCV_ = hypercellular tumor volume; V_p:_= fractional plasma volume; V_e:_= fractional volume of extravascular extracellular space.

**Table 4:** Summary of Diffusion-tensor imaging (DTI) techniques in brain cancer radiotherapy planning.

**#**	**Reference**	**Study Type**	**Functional MRI** (sequence, parameters & acquisition time)	**MRI-derived quantitative metrics**	**qMRI post-processing availability**	**Threshold** (range)	**Modification to treatment planning**	**Findings of changing radiation dose/target volumes**	**Technical limitations**
**6**	[29]	NR	**DTI** (SS-SE EPI; b = 0, 318, 392.5, 785, 1177.5 and 1570 s/mm^2^; 12 directions)	FA maps	In-house MATLAB	NR	**Standard plan** CTV = GTV + 2.5 cm **Theoretical DTI-based plan** CTV1 = IHV_DTI_ + GTV + 1 cm	1. IHV_DTI_ in CTV is smaller than standard CTV, PTV_DTI_ reduced size by a mean of 35%, resulting increasing doses mean of 67 Gy without increasing NTCP compared to PTV_standard_ = 60 Gy.	NR
**7**	[30]	NR	**DTI** (SS-SE EPI; b = 1000 s/mm^2^; 21 directions, 4 min)	Tractography and p & q maps	Syngo workstation and MATLAB	^1^NA	**Standard plan** CTV = GTV + T2W + 15–25 mm PTV = CTV + 5 mm (isotropic margin) **DTI-modified plan** CTV_DTI_ = GTV_DTI_ + pq map + tractography PTV_DTI_ = CTV_DTI_ + 2 cm (along WM tracts)	1. CTV_DTI_ smaller volume than T2W CTV & PTV_DTI_ show reduction in volume and irregular shape but not significant. Constrain dose to uninvolved brain tissues. Diffusion volumes < conventional volumes.	Geometrical uncertain tracts resolution
**8**	[31]	NR	**DWI**(SS-SE EPI)	Tractography 3D-FT maps	dTV software	^2^NA	**3D-CRT (SIB)**CTV1 = perifocal edema + 15-mm CTV2 = T1-CE PTV1 & PTV2 = CTVs + 5-mm	1. Dose reduction in CSTs and other OAR by integrating DTI into IMRT planning. Constrain max dose to eyeballs, optic pathways, brain stem and CSTs.	Different FT factors needs validation
**9**	[32]	NR	**DTI** (SS-SE EPI; b = 1,000 s/mm^2^; 25 directions; 280 s) **BOLD-fMRI** (EPI; Somatosensory tasks [finger tapping with audio cue])	Tractography and Colour-coded FA maps	MATLAB andMR advantage workstation.	^3^NA	PTV1 = 50 Gy; PTV2 = 60 Gy **IMRT** Not consider PMCs & CSTs **IMRT_PMCs&CSTs (fMRI + DTI)** Consider bilateral PMCs & CSTs	1. Dmax and Dmean decreased to bilateral PMCs and CSTs regions n IMRT_PMCs&CSTs on fused DTI and fMRI activation maps.No changes on PTV coverage but limiting dose to OAR is better than other plans.	NR
**10**	[33]	Prospective	**DTI** (b = 0, 1000 s/mm^2^; 32 directions)	DTI derived growth models	Anisotropy weighting parameter set to (y0) & (y20)	NA	**Standard plan** CTV_standard_ = GTV + 2 cm **Geometrical DTImodel plan:** CTV_DTI_ = CTVy0 & y20 normalized to CTV	1. CTV_DTI_ growth models have higher surface areas by 74% and 72% of CTV_standard_ were included in CTVy0 & CTVy20, respectively. Median surface area of CTV_standard_ = 211 cm^2^, CTVy0 = 338 cm^2^ and CTVy20 = 376 cm^2^	DTI parameters values need validation
**11**	[34]	Retrospective	**DTI** (SS-SE EPI, parallel imaging) **3 T:** (b = 0, 1000 s/mm^2^; 32 directions; 10.46 min) **1.5 T:** (b = 0, 1000 s/mm^2^; 15 directions; 12 min)	Mean diffusivity, FA maps, and Tractography	tracking algorithms	^4^FA of tracking = 0.1	**Original plan** CTV = GTV + 2 cm, PTV = CTV + 0.5 cm **DTItract-optimized plan (tractography)** volumes concerning of WM fibres prescriptions, TVs and OAR sparing.	1. Preserve PTV coverage by reducing WM tracts doses (SLF, IFOF, UNC and CSTs) Dose sparing was more relevant for contralateral tracts < 30 Gy	Susceptibility- geometrical distortions
**12**	[35]	Retrospective	**DWI** (NR)	DTI map DTI-FT for CSTs	standard deterministic algorithm	^5^NA	**Original plan** PRV not consider **VMAT plans for PRV-FT_TMS_** reducing dose to PRV-FT_TMS_ segments within the 30 Gy-IDL beyond the PTV	1. Reduce brain and OAR dose after CSTs sparing using Dmean < 6 Gy, to meet dose constraints. PRV-FT_TMS_ Dmean fell by 17.1%, decreased from 25.5 Gy to 21.2 Gy without PTV coverage changes.	NR


**Abbreviation:** 3D-CRT= three-dimensional conformal radiotherapy; 3D-FT maps= three-dimensional fibre tracking maps; BOLD-fMRI= Blood-oxygen-level-dependent imaging functional MRI; CSTs= corticospinal tracts; CTV_DTI=_ clinical target volume based on DTI; CTV= clinical target volume; CTVy0= the tumor cell density isoline encompassing the same total volume as the CTVstandard, with γ as an anisotropy weighting parameter γ set to =0 the diffusion is the same in all directions (isotropic); CTVy20= γ as an anisotropy weighting parameter set to =20 cell migration along the WM fiber tracts is higher than water diffusion; DTI= Diffusion tensor imaging; Dmax= dose maximum; Dmean= dose mean; DWI= diffusion weighted imaging; FA maps= Fractional anisotropy maps; fMRI= functional magnetic resonance imaging; FT= fibre tracking; GTV= gross target volume; Gy= gray (IS unit); IDL= isodose line; IFOF= inferior fronto-occipital fascicle; IHV_DTI_= image-based high-risk volume for abnormalities based on DTI; IMRT= intensity modulated radiotherapy; MRI= magnetic resonance imaging; NA= not applicable; NR= not reported; NTCP= normal tissue complication probability; nTMS= Navigated transcranial magnetic stimulation; OAR= organs at risk; p= Isotropic (p) map; PMCs= primary motor cortices; PRV-FT_TMS_= planning risk-volume fibre tracking of corticospinal tracts for transcranial magnetic stimulation; PTV= planning target volume; PTV_DTI=_ planning target volume based on DTI; q= Anisotropic (q) map; ; qMRI= quantitative MRI; SC= surgical cavity; SIB= simultaneous integrated boost; SLF= superior longitudinal fascicle; SS-SE EPI= single-shot spin echo echo - planar imaging sequence; T1-CE= T1-weighted contrast-enhanced; T_2_W= T2-weighted; UNC= uncinate fascicle; WM= White matter; VMAT= Dual volumetric arc IMRT.

**(1**) FA of seeding= 0.10, step size=0.5 mm, stopping criteria of FA= 0.1 and deflection angle= 60°; **(2)** stopping tracking of FA < 0.18; **(3)** FA of FT = 0.2, tract angular change= 30°; **(4)** stopping criteria of FA= 0.1, turning angle > 55°; **(5)** minimal fibres length of 100 mm and FA threshold of 0.1–0.

**Table 5:** Summary Magnetic resonance spectroscopic imaging (MRSI) techniques in brain cancer radiotherapy planning.

#	Reference	Study type	Functional MRI (sequence, parameters & acquisition time)	MRI-derived quantitative metrics	qMRI post-processing availability	Threshold (range)	Modification to treatment planning	Findings of changing radiation dose/target volumes	Technical limitations
13	[36]	NR	**3D-CSI MRSI** (PRESS; TE = 144 ms; matrix = 12 × 12 × 8 cm or 16 × 8 × 8 cm)	CNI CrNILLI	Interactive image analysis program	CNI ≥ 2	**3D-CRT** CTV = T2 + 1.5–2 cm + CNI2 **IMRT** CTV = T2 + 1 cm + CNI2	1. Metabolic imaging can determine residual tumours after resection. 2. Increasing CTV by 21 mm CNI2 outside T1-CE and RC. 3. Other metabolites were not definitive.	• Higher field for greater metabolic details. limited MRSI coverage.
14	[37]	Retrospective	**3D-MRSI** (PRESS; TE = 144 ms; matrix = 12 × 12 × 8 cm or 16 × 8 × 8 cm)	CNI	NR	CNI ≥ 2	**Conventional plan**GTV= (T2 + 1 cm) +/- (T1-CE + 2–3 cm) + RC **MRSI plan** GTV = GTV_conventional_ + CNI2	1. CNI2 exceeded 60 Gy IDL. 2. Nonuniform tumour margins reduced TVs and irradiated uninvolved normal brain tissue.	• limited MRSI coverage.
15	[38]	Prospective	**3D-CSI MRSI** (TE = 135 ms, matrix = 16 × 16, 8 min)	CNI	Siemens app Syngo MR B17 Spectroscopy	CNI ≥ 2	**60-Gy 3D-CRT (conventional) & IMRT** GTV1 = T1–CE **72-Gy SIB-IMRT**GTV2 = CNI ≥ 2	1. 72-Gy SIB-IMRT and 60-Gy IMRT increased TV dose, decreased max dose to brainstem, and reduced normal brain high dose-volumes (V36, V50) compared to 60-Gy 3D-CRT.	NR
16	[39]	Retrospective	**Whole-brain 3D-MRSI** (64 × 64 × 32 voxels)	MTV_Cho_ MTV_NAA_	MIDAS package	NR	**Standard plan** GTV = T1-CE CTV_46_ = T2/FLAIR + 2 cm (46 Gy) CTV_60_ = GTV + 2–2.5 cm + 14 Gy (60 Gy) **MRSI-derived MTVs plan** MTV_Cho_ & MTV_NAA_ to GTV, CTV_46_ & CTV_60_	1. MTV_Cho_ is 93%, 25%, 16%, and 3%, larger than GTV, edema, CTV_60_, and CTV_46_, respectively. 2. MTV_NAA_ is almost entirely contained within edema.	• Inhomogeneity area limit spectral quality Long acquisition times.
17	[41]	NR	**3D-MRSI (2HG imaging)**(TE = 68 ms, matrix 10 × 10 × 10, 9.55 min)	2HG/hCr ratio	LCModel software	2HG/hCr > 0.13	**Volumes of 2HG/hCr vs FLAIR** GTV = FLAIRCTV = GTV + 20–25 mm vs CTV + 2HG/hCr	1. 47% of patients had larger CTV of 2HG/hCr volume than FLAIR volume.	• limited coverage, data quality and spatial resolution.
18	[40]	NR	**Whole-brain 3D-MRSI**(19 min)	Cho/NAA ratios	MIDASMATLAB	Cho/NAA ratios = 1.5, 1.75, and 2.0	**Conventional plan** CTV1,2 = GTVs + 0.7 cm/0.5 cm PTV1,2 = 51 Gy, 60 Gy **MRSI-modified plan**CTV2_MRSI = CTV2 + Cho/NAA maps	1. CNI increase in volume beyond 60 Gy IDL for each threshold. 2. CNI CTV higher CE at recurrence coverage than conventional CTV. 3. <54 Gy max dose to the brainstem, optic chiasm, and optic nerves.	• Magnetic susceptibility affects spectral profiles.
19	[42]	NR	**Whole-brain 3D-MRSI**(TE = 50 ms; 15 min)	CNI	MIDAS BrICSb	CNI > 2compared to NAWM	**Conventional plan** GTV1 = FLAIR, PTV1 = 51 Gy GTV2 = T1-CE + RC, PTV2 = 60 Gy **MRSI plan**GTV3 = CNI ≥ 2 + T1-CE, PTV3 = 75 Gy	1. CNI volume is 50.6 cm^3^ larger than standard. 2. OAR dose constraints. 3. BrICS implemented MRSI to RT planning successfully	NR

Abbreviations: 2HG/hCr = ratio 2-hydroxyglutarate over healthy creatine; 2HG = 2-hydroxyglutarate; 3D-CRT = three-dimensional conformal radiotherapy; 3D-CSI = three-dimensional chemical shift imaging; BrICS = brain imaging collaboration suite: a cloud platform to integrates MRSI with clinical MRI volumes; Cho/NAA = a ratio of choline and N-acetylaspartate; CNI = Choline/N-acetylaspartate index; CNI2 = CNI at level 2; CrNI = creatinine/N-acetylaspartate index; CTV = clinical target volume; CTV 60 & 46 = at dose 60 Gy and 46 Gy; FLAIR = fluid attenuated inversion recovery; GTV = gross target volume; IDL = isodose line; IMRT = intensity modulated radiotherapy; LLI = lactate/lipid index; MIDAS = metabolite imaging and data analysis system; MTV_Cho_ = metabolic tumour volumes of high Cho (metabolite-defined treatment targets); MTV_NAA_ = metabolic tumour volumes of low NAA (metabolite-defined treatment targets); MRI = magnetic resonance imaging; NAWM = normal-appearing white matter; NR = not reported; OAR = organs at risk; PRESS = point-resolved spectroscopy sequence; PTV = planning target volume; qMRI = quantitative magnetic resonance imaging; RC = resection cavity; RT = radiotherapy; SIB = simultaneous integrated boost; T1-CE = T1-weighted contrast enhanced; T2 = T2-weighted; TE = echo time; TVs = target volumes.

### 3.3 DWI and PWI techniques

Table 3 describes detailed outcomes for five studies employing DWI/PWI techniques to modify brain cancer radiotherapy/SRS planning. Each study employed various sequences, parameters, and acquisition times. DWI was used alone to optimise dose for HGG RT planning [24], and in SRS planning for brain metastases [25] in an effort to reduce tumour recurrence. Both studies quantified ADC cellularity mapping using DWI at b-values of 0 and 1000 s/mm^2^. In these two prospective clinical studies, a combination of high b-value DWI, DCE-MRI and T1 quantification was used to guide RT planning [26], [27]. Although [27] is considered an interventional study with promising outcomes, the patients were not selected randomly, which may affect the strength of the results. In this study [26], DCE-MRI was quantitatively analysed to generate a high CBV (hCBV) map using a three-parameter Tofts model from T1 mapping to correct non-linear signal response to contrast agent concentration. High b-value DWI images (3000 s/mm^2^) were utilised in this study to contour the hypercellular volume (HCV) non-quantitatively, without calculating quantitative ADC metrics. Another study by Brighi et al. [28], examined the repeatability of RT dose-painting prescriptions maps using DWI with (b-value = 0 & 700 s/mm^2^) and DSC-MRI models (CBV and CBF).

One study reported a 15-fold increase in dose conformity to the high-risk clinical TV (CTV_HR_) based on average ADC compared to the conventional plan [24]. Another study showed that combining HCV and hCBV for treatment planning reduced target volume size by 1.8 times compared to T1-contrast enhanced (T1-CE) and by ten times compared to fluid attenuation inversion recovery (FLAIR), resulting the mpMRI boost volumes [26]. However, this paper was included in this review based on quantitative perfusion maps combined with HCV/hCBV. Kim et al. [27] reported in this only interventional study that the use of dose-intensified RT in regions of hypercellular tumour volume (TV_HCV_) and hyperperfused tumour volume (TV_CBV_), the median of overall survival (OS) and progression-free survival (PFS) was 1 month longer than an overall cohort. In contrast, TV delineation based on volumes from combining ADC and T1-CE TV would define a significantly greater metastasis treatment volume than obtained on standard T1-CE volume alone [25]. A recent repeatability study provides a technical validation of a practical framework to generate dose-painting prescriptions and tumour probability maps on mpMRI models by combining DWI and perfusion parameter metrics for reliable dose-painting RT planning [28].

### 3.4 DTI techniques

Table 4 summarises outcomes from seven DTI studies conducted for HGG and GBM target delineation for RT planning. In one study, patients were selected who had brain lesions near to primary motor cortices (PMCs) and corticospinal tracts (CSTs) [32], whereas the other study retrospectively selected patients who had lesions near CSTs and undergone preoperative navigated transcranial magnetic stimulation (nTMS) motor mapping [35].

In all studies, quantitative DTI maps were measured using a variety of diffusion gradient directions and magnetic strengths to reconstruct DTI maps. A study by Jena et al. [29] used thresholds to FA maps to show WM tract distortion due to tumour infiltration. Similarly, Berberat et al. [30] calculated FA, (q) and (p) diffusion maps to identify tumour infiltration expanding the CTV and planning target volume (PTV) isotopically and anisotropically along diffusion tensor tracts maps. Another study used directional diffusion data to simulate the tumour growth modelling by defining tumour cell density, as an alternative way to grow CTV from gross target volume (GTV), rather than using isotropic margins [33].

Conversely, a tractography map can generate WM tract models based on FA values and other fibre-tracking (FT) factors that are vary across studies. This was addressed by Igaki et al. [31], for CST sparing using 3D-FT maps created from tractography, and multiple WM fibre bundles have received reduced mean doses by reconstructing tractography and calculating mean diffusivity (MD) and FA maps [34]. Only one study integrated BOLD-fMRI and DTI to spare PMC and CST fibers located adjacent to the TVs [32]. This study employed tractography and colour-coded FA maps from DTI and activation maps from BOLD-fMRI. Diehl et al. [35] assessed the use of functional mapping with nTMS along with DTI-FT, for CST delineation for dose reduction.

All studies reported dosimetric and volumetric modification outcomes to optimise RT planning. Two studies demonstrated reductions in TVs with increased TVs doses and/or similar or reduced OAR and normal brain tissue doses [29], [30]. While other study observed increases in the CTV_DTI_ surface area volume with radiation dose changes using growth-models DTI maps [33]. The remaining studies also showed reductions in the maximum dose and sparing of WM fibre tracts and other OAR without TVs changes [31], [32], [34], [35].

### 3.5 MRSI techniques

Table 5 summarises findings for 3D-MRSI in brain cancer RT planning. These studies [36], [37], [38] reported assessments of proton (^1^H) 3D-chemical shift imaging (CSI) MRSI utilising long echo times (TEs) of 135–144 ms rather than short TE in 1.5 T scanners to obtain additional spectral information. Additionally, three studies [39], [40], [42] used ^1^H whole-brain volumetric 3D-MRSI in 3.0 T scanners, as this allows for better metabolic volume acquisition of the entire brain region. However, Jafari et al. [41] applied a new 3D-MRSI sequence rather than ^1^H MRSI, a 2-hydroxyglutarate (2HG) signal to detect mutated isocitrate dehydrogenase-1 (IDH1) gliomas in the brain for target extent definition and comparison to FLAIR images.

Five studies quantified MRSI data to assess tumour infiltration on a voxel-by-voxel basis. Cho/NAA index (CNI) with a threshold ≥ 2 indicated a brain tumour metabolic abnormality. One study utilised metabolic tumour volumes of low NAA (MTV_NAA_) and high Cho (MTV_Cho_) then normalised the Cho magnitude with water signal to identify tumour activity and infiltration compared to standard RT volumes [39]. Another approach is to use 2HG imaging to provide an optimal threshold to differentiate between tumours and normal regions by comparing the 2HG signal to the mean value of healthy creatine (2HG/hCr map) with a cut-off value > 0.13 [41]. All included studies integrating metabolic imaging showed increases in GTV or CTV coverage based on CNI or 2HG/hCr volumes, beyond the standard TVs, with nonuniform margins for tumour infiltration regions. Only three studies reported reduced OAR doses and improved TVs doses by integrating MRSI into RT planning [38], [40], [42]. Several technical challenges, such as, magnetic field strength, limited MRSI coverage, low spectral quality and longer acquisition time were investigated in some studies.That said, amany advances in MRSI techniques still remain highly challenging in terms of consistency in implementation for brain RT planning. However, a cloud platform called Brain Imaging Collaboration Suite (BrICr) has been developed to facilitate integrating MRSI into RT planning workflows to improve tumour targeting and delineation across multi-institutions [42].

## 4 Discussion

This work presents the first comprehensive systematic review regarding the integration of quantitative MRI biomarkers to optimise brain tumour RT planning. Four main functional MRI techniques were identified and categorised into three main groups (DWI and PWI, DTI, and MRSI) with key features summarised for each group. This review investigated the clinical evidence for using these quantitative MRI biomarkers to improve RT planning.

MRI-based RT planning enhanced brain cancer TVs and OAR delineation and facilitated dose escalation, resulting in significant differences in GTV and CTV [3]. DWI and PWI techniques evaluate hypercellularity and hyperperfusion to detect tumour microenvironment heterogeneity that cannot be seen with conventional MRI. Quantitative mpMRI imaging biomarkers have shown potential in evaluating therapeutic response, prediction, tumour differentiation and grading [4], [5], [7]. For example, acquiring the parametric response map of ADC and rCBV at week three of treatment was considered a strong predictor of early cancer treatment response in HGG patients [43]. ADC value is calculated using two or more b-values to assess the level of diffusion weighting. The highest b-values in studies varied from 700 to 3000 s/mm^2^. Combining HCV with quantitative hCBV derived from DCE permits targeting of smaller GTV tumour subvolumes than T1-CE and FLAIR by mean volumes (13.1 compared to 23.9 and 128.9 cm^3^, respectively) [26].

As highlighted in the five studies [24], [25], [26], [27], [28], ADC can be used alone or in combination with perfusion metrics to help delineate brain tumour volumes to optimise RT doses. A study indicated that dose escalation can be improved by identifying ADC-based CTV_HR_ while sparing OAR [24]. However, a relative ADC threshold in [24] was calculated from just one patient’s data; therefore, this threshold might vary if more patients were included. This study included the lowest number of participants compared to the other studies in this category (ranging 11–38 patients) resulting in less generalisable results. Although [26], [27] included 38 patients in total cohorts were not matched in all aspects of age and MGMT promoter methylation status. Zakaria et al. [25] found that combining ADC and T1-CE resulted in larger GTV contours, led to a reduction in the volume of recurrence compared to using T1-CE alone in conventional MRI planning. While Kim et al. [26] defined CTVs were smaller for HCV and hCBV than T1-CE and FLAIR volumes which help boost volumes for dose-intensified RT. Evidence from Kim et al. Interventional study [27] supported the findings of the previous cohort [26] that the dose escalation to TV_HCV_/TV_CBV_ may yield promising survival outcomes_._ The repeatability of fusing mpMRI models (ADC and rCBV map, and ADC and rCBF map) was also assessed by generating relative differences in the quantitative metrics to boost doses in tumour infiltration areas to improve RT plans [28]. Differences in tumour characteristics; MRI acquisitions and scanners; and RT planning types across studies complicated comparisons of the clinical evidence.

The full DTI map can calculate multiple quantitative metrics, including FA maps that can describe directional diffusion and show orientations by obtaining FA colour-coded maps. Mean diffusivity (MD) maps measure the average magnitudes of diffusivities in WM microstructure at a particular voxel; while, isotropic (p) and anisotropic (q) diffusion maps evaluate tumour infiltration. All these maps can be thresholded [12]. One study indicated that post-radiation tumour necrosis could be differentiated from recurrence through changes in reported lower FA and higher MD values [4]. FA maps can be evaluated to assess WM fibre tract displacement, disruption or infiltration due to tumours. A full DTI map can also be analysed to either generate WM fibre tracts models (“tractography”) or using complex modelling processes such as tumour-growth simulation [13]. Variations of FT parameters values may impact the clinical significance, which requires optimization and validation.

DTI is sensitive to defects in WM tracts caused by mass effects or oedema that correlate with tumour infiltration; these defects cannot be identified using anatomical MRI [44]. DTI has been combined with a functional neuronavigation system to improve preoperative CST location assessment to facilitate avoidance during surgery [45]. A diffusion-proliferation model based on DTI data has also been developed to incorporate anisotropic diffusion of tumour growth to fit the lesion shape accurately since glioma cells preferentially infiltrate along WM tracts [46]. This approach has been used to modify TV size in RT planning [30] based on tractography maps and infiltration maps, including p and q diffusion maps, based on an FA of FT threshold of 0.1. A reduction in CTV_DTI_ and PTV_DTI_ coverage were demonstrated compared to conventional CTV, while sparing the normal brain tissue. Similarly, Jena et al. [29] reduced PTV_DTI_ volumes by a mean of 35%, by defining CTV on DTI high-risk volumes.

Reducing maximum doses to OAR is critical for limiting radiation injury risk to normal tissues [47] and may be facilitated using non-uniform CTV margins [48]. A retrospective dosimetric planning comparison of multiple bilateral WM tracts demonstrated reductions in radiation doses with DTI integration [34]. Similarly, reductions in mean doses to WM tracts and other OAR, without affecting CTV and PTV coverage volumes, was achieved by incorporating DTI alone [31], DTI and fMRI [32] or preoperative nTMS and DTI [35]. However, long-term randomised clinical trials (RCTs) are needed to confirm the clinical benefits, such as reductions in neurological injury, of dose sparing to WM tracts. These findings support the conclusions of a recent systematic review that assessed the benefits of implementing DTI for WM dose optimisation in clinical practice [21]. The results showed that DTI enhanced sparing of critical WM tracts without affecting volume coverage of the tumour, but DTI acquisition parameters needed optimisation and standardisation.

The technical limitations of DWI and DTI techniques lie primarily in using echo planar imaging (EPI) sequences, as these are prone to geometric distortion artefacts induced by field inhomogeneities [49]. This distortion results in voxel shifting, which may affect FT outcomes and the accuracy of identified tumour margins if using DWI and DTI for RT planning. Different distortion correction techniques, such as readout segmented EPI (RESOLVE) and parallel imaging techniques, are available to minimise image distortion [50]. These techniques have been used in two studies [26] and [34] during imaging acquisitions. Studies that do not use these strategies may experience geometric distortion, that impact clinical outcomes. Geometric distortion remains a concern in clinical practice, therefore, quality assurance tests are recommended to improve geometric accuracy [51].

MRSI is a valuable tool for detecting Cho/NAA abnormalities when examining residual disease after surgical cavity resection, thus facilitating non-uniform margins [52]. Routinely, T1-CE or T2/FLAIR images may display enhanced necrotic/oedema areas or miss the nonenhanced active tumour tissues within TV. Gliomas show increases in Cho/NAA ratios when correlating metabolite levels with histologic findings of image-guided surgical biopsies [53]. Using MRI molecular imaging methods rather than conventional MRI in RT planning improves the target definition of microscopic tumour infiltration [54]. Thus, metabolite concentrations provide reliable quantitative MRSI data for detecting tumour extension beyond conventional 60-Gy isodose lines [37], [40]. A longitudinal prospective study showed that MRSI could predict GBM relapse sites post-RT [55], therefore, this technique could be used to boost metabolically abnormal subvolumes (e.g., Cho/NAA set abnormality index (AI) to ≥ 2).

Additional spectral distribution information about metabolites concentrations, such as lactate and lipid, can be acquired using long TEs [56], which are indicators of tumour grade, necrosis and hypoxia [57]. The results for the CNI threshold across studies are consistent, with AI ≥ 2 to detecting metabolically abnormal volumes and margins. Interestingly, all seven studies [36], [37], [38], [39], [40], [41], [42] integrating MRSI into RT planning reported increased GTV or CTV volumes beyond the conventional TV, leading to improved dose escalation while sparing OAR. A recent ongoing dose-painting trial in newly diagnosed GBM patients found CNI ≥ 2 to improve tumour definition for dose escalation, while also using other multimodal MRI, including DWI and DSC [58]. Another approach using MRSI-based 2HG was established to show potential for detecting tumour-specific metabolites [59]. This technique was employed as an imaging biomarker of IDH1-mutated glioma and showed greater CTV volume using 2HG/hCr volume, but it still presented many technical challenges [41].

Most abnormal metabolic activity is located beyond conventional TV. Previously, MRSI acquisition had a restricted scanning matrix size, leading to missed detection of microscopic tumour infiltration. To overcome this limitation, whole-brain volumetric 3D-MRSI was developed and included the entire brain volume to evaluate metabolic maps in affected areas. A first study used whole-brain MRSI conducted by Parra et al. [39] found that increased volume coverage led to accurate identification of TV with better representation of metabolically active brain regions. The signal-to-noise ratio (SNR) for metabolite quantification is better with higher field strength (3 T) MRI scanners than with 1.5 T scanners. Cordova et al. [40] developed a pipeline of high resolution, whole-brain 3D-MRSI acquisitions and analyses for RT planning by overcoming a number of technical limitations of limited coverage, time consumption and lack of post-processing protocol.

The potential of using MRSI prospectively in guiding RT planning needs further clinical practice investigations due to the lack of standardisation of MRSI acquisitions and spectra analysis processing. Some limitations, including long acquisition times (>10 min), which may degrade spectral quality since motion and related artefacts may be more likely. Poor MRSI shimming from lipid contamination must be avoided by applying spatial selective saturation bands around the skull [60]. Different vendors have addressed technical complexities by integrating 3D-MRSI data post-processing into RT planning systems. A multi-institution pilot study was conducted to develop a BrICS cloud platform to define TV between institutions and then import MRSI data into the RT planning systems [42].

The results of the present work suggest that DWI and PWI can be better used for determining TV coverage for dose escalation than for sparing OAR. Both play a significant role in oncologic applications; however, the five included studies showed various outcomes for improving RT planning because of insufficient evidence for standardised relative threshold values of ADC and perfusion maps. Integration of DTI into RT planning is considered a promising technique, whereas delineating WM fibre tracts close to a tumour can effectively spare OAR and healthy brain tissues from a high dose. Although DTI may not affect TV delineation, the dose distributions may be improved compared to conventional RT planning. Nevertheless, DTI lacks a standard validation technique because of several factors, including diffusion directions, MRI scanner features, diffusion metrics biomarkers, reconstruction algorithms, ROI location, size for tract seeding and FA thresholds, impact FT analyses. The most prominent finding is that MRSI metabolic maps can show heterogeneity within a tumour and can help to produce a non-uniform dose distributions plan for better treatment of brain lesions. These metabolic maps can effectively identify tumour infiltration based on high CNI abnormality, thereby allowing a boost in dose to that region while sparing the surrounding normal tissue. Many technical considerations, such as using 3 T MRI for better SNR and optimisation shimming, should be taken into account to improve spectral quality. Overall, the results identify two promising techniques: DTI and MRSI; however, DWI and PWI results were not particularly encouraging, as they were not consistent, and the four studies presented different positive outcomes.

This review found no MRI-based radiomics studies for RT planning to improve treatment of brain cancers; radiomics may not be ready to be implemented clinically due to a lack of clinical validation of MRI techniques and parameters.

The included studies had many limitations, including the small sample size (ranging from 1 to 38 patients between studies) that could affect generalisation of clinical evidence. These studies require clinical validation with long-term follow-up. Additionally, with the exception of the only interventional study [27] which was not a randomised trial, the included studies were single centre in silico planning or retrospective replanning studies, which have fewer clinically relevant outcomes. Unfortunately, the lack of prospective RCTs raise uncertainty about the clinical impact of quantitative MRI biomarkers in improving RT planning. Theoretical RT planning, such as DTI studies, that include only lesions that are close to WM fibres may raise concerns regarding selection bias. Adding to these limitations, MRI acquisition techniques are varied among studies that used similar technique due to the use of different MRI scanners, magnetic field strengths, post-processing analyses and means of importing quantitative MRI data into RT planning systems. Using 3 T MRI scanners would improve MRSI quality and DTI measurements but may be more susceptible to distortion at higher field strengths. The longer acquisition times for acquiring advanced techniques images may also impact clinical workflow. Current practice variations might complicate clinical implementations of advanced MRI techniques.

Further multi-centre prospective RCTs with clinical endpoints are recommended to provide a meaningful conclusion on the clinical benefit and feasibility of advanced MRI techniques to radiotherapy outcomes. Combining different quantitative MRI biomarkers may improve RT planning outcomes in terms of volume/size of TV and facilitating dose escalation. Integrating mpMRI-based radiomics could also improve target definition. Standardising MRI acquisition parameters and quantitative metrics is essential for clinical implementation between advanced MRI techniques and vendors; therefore, consensus guidelines are needed. A meta-analysis would be beneficial for comparing quantitative analyses across studies using similar MRI techniques and methods but with different outcomes for thresholds or acquisition parameters. In conclusion, although a range of imaging techniques is available for potential use in modifying RT planning approaches, the existing studies in this review are mostly planning studies and lack associations with the resulting clinical outcomes. Limited studies have been conducted to investigate the correlation between treatment volumes and outcomes. Multi-centre RCTs with clinical endpoints are still required to confirm the clinical benefits.

Quantitative MRI biomarkers hold promise for improving RT planning. Integrating these techniques may enhance the accuracy of high-risk TV delineation and facilitate dose escalation while sparing OAR. DTI shows potential for facilitating OAR sparing, but further clinical evidence with optimised parameters is needed. MRSI is feasible for clinical practice, and the Cho/NAA index improves target delineation of tumour infiltration for dose escalation without increasing OAR dose, but technical considerations should be addressed. Evidence is insufficient to draw conclusions about the value of employing DWI and PWI in RT planning, but various positive outcomes are evident among the studies. Consensus guidelines are needed to optimise technical parameters for MRI techniques and these should then be evaluated in RCTs.

## CRediT authorship contribution statement

**Abeer M. Aldawsari:** Writing – original draft, Conceptualization, Methodology, Investigation, Visualization. **Bashar Al-Qaisieh:** Writing – review & editing, Conceptualization, Methodology, Investigation, Supervision, Project administration. **David A. Broadbent:** Writing – review & editing, Conceptualization, Methodology, Investigation, Supervision, Project administration. **David Bird:** Writing – review & editing, Conceptualization, Methodology, Investigation, Supervision, Project administration. **Louise Murray:** Writing – review & editing, Conceptualization, Methodology, Investigation, Supervision, Project administration. **Richard Speight:** Writing – review & editing, Conceptualization, Methodology, Investigation, Supervision, Project administration.

## Declaration of Competing Interest

The authors declare that they have no known competing financial interests or personal relationships that could have appeared to influence the work reported in this paper.
